# Motor Simulation without Motor Expertise: Enhanced Corticospinal Excitability in Visually Experienced Dance Spectators

**DOI:** 10.1371/journal.pone.0033343

**Published:** 2012-03-21

**Authors:** Corinne Jola, Ali Abedian-Amiri, Annapoorna Kuppuswamy, Frank E. Pollick, Marie-Hélène Grosbras

**Affiliations:** 1 School of Psychology, University of Surrey, Guildford, United Kingdom; 2 Brain Imaging Centre, McGill University, Montreal, Canada; 3 Department of Clinical Neuroscience, Imperial College, London, United Kingdom; 4 School of Psychology, University of Glasgow, Glasgow, United Kingdom; 5 Institute of Neuroscience and Psychology, University of Glasgow, Glasgow, United Kingdom; University of Reading, United Kingdom

## Abstract

The human “mirror-system” is suggested to play a crucial role in action observation and execution, and is characterized by activity in the premotor and parietal cortices during the passive observation of movements. The previous motor experience of the observer has been shown to enhance the activity in this network. Yet visual experience could also have a determinant influence when watching more complex actions, as in dance performances. Here we tested the impact visual experience has on motor simulation when watching dance, by measuring changes in corticospinal excitability. We also tested the effects of empathic abilities. To fully match the participants' long-term visual experience with the present experimental setting, we used three *live* solo dance performances: ballet, Indian dance, and non-dance. Participants were either frequent dance spectators of ballet or Indian dance, or “novices” who never watched dance. None of the spectators had been physically trained in these dance styles. Transcranial magnetic stimulation was used to measure corticospinal excitability by means of motor-evoked potentials (MEPs) in both the hand and the arm, because the hand is specifically used in Indian dance and the arm is frequently engaged in ballet dance movements. We observed that frequent ballet spectators showed larger MEP amplitudes in the arm muscles when watching ballet compared to when they watched other performances. We also found that the higher Indian dance spectators scored on the fantasy subscale of the Interpersonal Reactivity Index, the larger their MEPs were in the arms when watching Indian dance. Our results show that even without physical training, corticospinal excitability can be enhanced as a function of either visual experience or the tendency to imaginatively transpose oneself into fictional characters. We suggest that spectators covertly simulate the movements for which they have acquired visual experience, and that empathic abilities heighten motor resonance during dance observation.

## Introduction

It has been suggested that observers internally simulate other peoples' actions [1] through neurons located in distinct frontal, motor, and sensory cortices that fire both when observing and executing movements [2]–[3]. The automatic response of these neurons in the so-called “mirror network” to passively observed actions is suggested to allow the beholder to understand the meaning of those actions by means of internal simulation [4].

Many studies have shown that the motor expertise of the observer enhances this simulation process in a kinematically congruent manner [5]–[7]. For example, activity in the brain regions involved in action observation is increased in spectators who are physically trained to execute the observed movements relative to spectators who are less experienced in executing these movements [8]–[11]. Furthermore, the activity in parts of these brain areas (inferior parietal lobe and ventral premotor cortices) is enhanced relative to how confident the spectators feel in executing the movements, even after only a short learning period [12]–[14]. Clearly, though, visual information also plays an important role in motor learning. Catmur et al. [15] found evidence that repeated incompatible visual feedback during motor execution altered the neuronal response to action observation. We are interested in finding out if repeated visual exposure alone can modify motor simulation.

About 40% of a typical dance audience have no physical experience of the movements they are keen to watch [16]. However, no studies have yet investigated how repeated visual exposure alone, in the absence of physical training, affects the neuronal processing that takes place when a person observes other people's movements. According to the direct matching hypothesis of the mirror-neuron theory, an action is understood when the motor system of the observer resonates with that of the performer's [17]. Indeed, a number of studies have found evidence that the mirror-neuron network activity is sensitive to the kinematics of the observed actions [see review in 18]. Thus, under the assumption of a direct matching between an observed action and the internal motor representation of that action, physical experience appears to be a pre-requisite for motor resonance. Clearly, physical training alters motor representation [e.g. 12], supporting the proposed link between action production and perception. Yet perceptual experiences alone have also been found to affect perceptual and/or motor processes. For example, it is known that visual observation leads to a significant improvement in motor learning [e.g. 19]–[20], with corresponding changes in neuronal activity [e.g. 21]–[22]. Also, two recent behavioral studies [10], [23] have found evidence that visual exposure can shape the perceptual processes involved in watching dance. Hence we suggest that visual experience in the form of repeated visual exposure alone may modify motor-related simulation processes at the neuronal level. For instance, over the years, an individual who loves watching ballet becomes visually experienced in watching that particular type of dance for the simple reason that he/she has - more likely than others - seen more ballet performances and has watched these with greater interest. Hence, the deliberate exposure to watching performances in specific dance styles may form a visual experience of a spectator that is different from the familiarity gained by the inevitable exposure to culturally coded visual stimuli in our everyday life. Visual experience, as studied here, could potentially lead to a motor mapping that is closely linked to the actual motor aspects of an action. We thus investigated whether ballet spectators - who deliberately expose themselves to ballet dance performances - show signs of motor simulation of ballet specific dance movements even though they have never been trained in ballet and therefore have gained visual but no physical experience.

One way to measure neuronal changes in the motor circuit is to probe, under different conditions, the primary motor cortex with transcranial magnetic stimulation (TMS) while recording the muscular response with electromyography (EMG, [see 24–25]). Larger motor evoked potentials (MEPs) indicate higher excitability of the corticospinal tract [see 26] at the moment of stimulation. Several TMS studies have shown that during the perception of actions, MEP amplitudes are modified in a phase-specific [27] and muscle-specific way [28]–[30]. This has been taken as an indication of motor simulation or motor resonance [see 31]. Using this method, Aglioti et al. [5] demonstrated that visual experience of watching basketball actions modulated corticospinal excitability during action observation, although – in line with the direct matching hypothesis – only basketball players showed fine-tuned modulation of the MEPs related to prediction of the outcome of basket shots. While physical and visual experience can be dissociated to a certain extent [see also 9], social factors that are inherently intertwined with either physical or visual experiences may interact and impact on motor corticospinal excitability in a complex manner [32]. For example, Molnar-Szakacs and colleagues [33] found that the corticospinal excitability of a spectator was enhanced when they observed culturally coded hand gestures performed by an actor of their own linguistic, regional or cultural community compared to the same movements performed by a member of another community. Here, we were interested in whether self-reported visual experience acquired by repeated exposure over the years with no physical knowledge of the movements observed can provide responsive fine-tuned muscle-specific internal motor simulation, in particular when the dance is situated within the spectators' cultural experience.

Dance offers unique stimuli that are well-suited to studying interindividual variability in motor system engagement during action observation, for three reasons: 1) The movement repertoire in dance is practically unlimited, hence dancers can produce a vast number of movements which provide them with unique motor or visual expertise. This then provides spectators with a practically unlimited number of visual experiences. 2) Dance has both formal and gestural movement vocabularies, and both can be independent of external objects and spatial locations, distinct from ball games or simple goal-oriented everyday actions [8]. As a result of this, individual differences in watching dance reflect the spectators' experience, not the objects or goals, as is the case with the grasping tasks that have previously been used to investigate the mirror-neuron system in humans [2], [17], [34]–[35]. 3) Dance is multicultural, multilayered, and multisensory, but several dance styles are defined by their specific kinematic motion patterns, types of music, costumes, lights, and stage settings; therefore, individuals may acquire knowledge of specific dance styles based on their experience of watching performances with *dance-style specific performers*, *costumes*, *music*, and *movements*. Supported by two studies that suggested different processing in sensorimotor brain areas when body movements were seen on a screen compared to live [36]–[37], with larger activity in the live condition, we argue that only by using a *live* performance with the corresponding *music* and *costumes* can we guarantee that we will capture the multifaceted aspects of watching a performance that the experienced dance spectator would normally encounter. As a large number of brain imaging studies, conducted under strict, controlled experimental conditions, have now provided us with a good understanding of the organization of the action observation network, we believe that it is time to complement this body of investigations with studies bearing a higher ecological validity [see 38].

To address the question of visual experience in specific dance movements, we chose to use two globally established but juxtaposed dance styles, namely ballet and Bharatanatyam [39], which use arms and hands differently. Indeed, according to the direct matching hypothesis, the modulation of TMS induced MEPs should be muscle specific: watching arm movements has been shown to enhance corticospinal excitability of the arm, but not hand, representation and vice –versa [30]. In both chosen narrative dance forms, the upper body parts are used throughout to communicate meaning to the spectator by the use of formal movements and gestural expressions, but the manner in which this meaning is conveyed differs notably between them. In Bharatanatyam, a classical Indian dance form, henceforth referred to as “Indian dance”, the basic technique is called ‘hasra mudras’, which refers to the different ways of using the hands and fingers for the gestures [40]. The specific aesthetic of ballet, which is a type of performance dance, henceforth referred to as “ballet”, can be recognized, amongst other features, by its five strictly defined arm postures [41], while the fingers are held in the same position throughout the dance. We thus expected enhanced responses in the forearm when experienced ballet spectators watched a ballet performance and in the hand when experienced Indian dance spectators watched Indian dance.

Our choice to study the corticospinal excitability of upper-body limbs is supported by a number of factors, beyond the practical reason that they are easily accessible to TMS using a standard circular coil. First, observers' responses to arm and hand actions have been studied widely to test the execution-observation matching system [e.g. 38]. These previous studies thus provide a useful point of comparison for our study. Second, the upper body is the most likely focus of attention for someone who is watching dance. Ballet is not a “collaboration of steps” as is often erroneously assumed; steps are a means of transferring the body centre, which itself is the core of ballet practice [42]. When watching a dance film, novices do focus more on the background near the upper body parts than the legs [23]. Even in everyday circumstances, men and women fixate first and foremost on the upper body, rather than the lower body [43]. And finally, the importance of the arms in dance is reflected in the intense training dancers undergo, leading to enhanced proprioceptive acuity of the arms of dancers compared with controls [44].

While our primary question related to the role of visual experience, given the complex nature of the performances and our different spectator groups' relationship to the artform, we also wished to explore how other personal factors might influence motor resonance while watching dance. The ability to project oneself onto the observed object of contemplation, as is the case when watching dance, is known as empathy. Indeed, empathy has been regarded as the most relevant factor in aesthetic appreciation [45]. In particular, enhanced empathic abilities in a healthy population were found to increase cortical excitability during action observation [46].

If dance spectators' motor simulation was intensified by their empathic responses, we would expect to find increased motor corticospinal excitability for spectators who score high on empathy compared to those who score low. Empathic responses could potentially modify corticospinal excitability to a greater extent than visual expertise.

One specific population we might consider here is people with autism spectrum disorder (ASD), who have low skills in social interaction, commonly associated with low empathic abilities. Indeed a study using the autistic quotient as a proxy for empathy reported that participants who scored high (i.e. with low empathic abilities) showed no significant modulation of corticospinal excitability when observing meaningful hand mouth actions compared to stills as found in participants who scored low [47]. Importantly though, the assumption that ASD is linked to low empathic abilities is not uncritical [48]–[49]. Clearly, however, emotional expressive stimuli significantly enhanced the response of a putative mirror-neuron system [50], [ but see 51]. We therefore expect empathic factors to affect corticospinal excitability more when watching dance styles that consist of direct and universal expressive gestures than when watching those that consist of formal and codified styles such as ballet.

To summarize, we used TMS to study the effects of visual experience and empathic ability on corticospinal excitability while watching a *live* dance performance. Based on the previous findings mentioned above, we expected the observers' visual experience and empathic skills to enhance motor simulation, as indicated by larger amplitudes in MEPs. We therefore measured participants' TMS induced MEPs while they viewed a *live* ballet performance, a *live* Indian dance and a *live* non-dance acting control condition. Participants were either frequent ballet or Indian dance spectators, or novices who never watched dance. None of the participants had been trained in any of these dance forms. Additionally, participants' levels of empathic abilities were measured and we also asked them to indicate their acquired visual experience in the dance performances used in this experiment.

## Methods

### Participants

Thirty-two healthy participants (between 20 and 72 years, 10 male) participated in the experiment. Only participants with no formal dance training were recruited. All participants were recruited with support from the Theatre Royal in Glasgow, Scotland's Indian Dance Company Ihayami, the MELA Indian dance festival organizers, and via the University of Glasgow advertising webpage. Participants were invited to participate in the study based on their responses to a telephone screening and to a custom-designed paper-and-pencil custom designed questionnaire. This was used to select the best candidates in terms of age, gender, dance type interest, and how many performances they intended to watch over the time-course of an average year. Since audience behaviors and subsequent reports are always influenced by their cultural and social competence, experience, and motivations, we asked for the amount of performances they intended to watch rather than the number of performances they were actually able to attend. In the screening, however, participants were further assessed on their dance experience. They were invited to participate in the study if they were able to confirm that they had had no formal dance training in any dance style and no form of training in either ballet or Indian dance. In addition, they had to fit into one of the three categories: novices, with no visual experience of watching any type of dance, or one of two types of visually experienced spectators, who had intended to watch at least five dance performances of either ballet or Indian dance per year over the last five years. The study was reviewed and approved by the local ethics committee (Ethics Board of the College of Science and Engineering at the University of Glasgow) with reference number FIMS0522. All participants gave written informed consent and received a small fee.

Three participants were excluded from the analysis: one female Indian dance spectator because she fell asleep during the performance, one female novice spectator did not show MEP responses during the experiment and one female ballet spectator did not participate in the whole study. Of the 29 participants with mean age 45.4 (16.8 SD), 12 (mean age of 52.4, SD 13.8) were experienced in watching ballet. Eight others (mean age 51.6, SD 17.5) were experienced in watching Bharatanatyam (a classical form of Indian dance). Nine novice participants (mean age 31.0, SD 10.0) had no experience in watching any form of professional dance. Ballet and Indian dance spectators did not significantly differ in age, *t*(18) = 0.11, *P* = 0.911. However, novices were significantly younger than Indian dance spectators, *t*(10.82) = 2.98, *P* = 0.039 (corrected for unequal variances) as well as significantly younger than ballet spectators, *t*(19) = 4.00, *P* = 0.004, all Bonferroni corrected independent *t*-tests at alpha 0.05 for three comparisons. We thus used age as a covariate of no-interest in the analyses of visual experience on corticospinal excitability. The proportion of females did not differ from equal distribution for any of the spectator groups as shown by one sample non-parametric binominal tests, *P* = 0.146 (ballet spectators, 9 female, 3 male), *P* = 0.727 (Indian dance spectators, 5 female, 3 male), and *P* = 1.000 (Novices, 4 female, 5 male). The majority of our participants were Caucasian and came either from the UK (19 out of 29) or from mainland Europe (4). Participants with black or mixed skin color were from South Africa (1) and India (5). The ballet and novice spectators were all from a white European background, while the Indian spectators were from India (5), the UK (2) or South Africa (1). Because differences in skin color and culture have been found to affect corticospinal excitability in action observation [32]–[33], we ran an additional analysis using skin color as a between-subjects factor.

### Stimuli

Three groups of participants with specific visual experience in watching dance (ballet spectators, Indian dance spectators, and novices, see above) passively watched three types of performances (ballet, Indian dance, and acting control condition). Each performance lasted five minutes and was performed by a professional female dancer or actor to recorded music. Further, a baseline rest condition was conducted where participants' corticospinal activity was measured while they relaxed and had their eyes closed.

The ballet performance was a concatenation of three fairy solos from the Royal Ballet version of Sleeping Beauty; ‘Breadcrumb Fairy’, ‘Enchanted Garden’, and ‘Lilac Fairy’, plus a mime section from the ‘Lilac Fairy’ role to the sound of ‘Crystal Fountain’. The choreography for the ballet performance was modified by the first author of this paper, who is trained in choreography, and was externally reviewed by the dance scholar Jang Seon Hee from Sejong University, Korea. The music played was extracts from Sleeping Beauty – Ballet in a prologue and three acts from Tchaikovsky Op. 66 (2007). The Indian dance was a Bharatanatyam piece, a popular ‘padam’ from the traditional repertoire describing the God Krishna's childhood pranks. The music was ‘Theeradha Vilayattu Pillai’ by ‘Subramanya Bharathiyar’ taken from Nupura Naadam. For the non-dance acting control piece without voice, an existing performance was modified in a collaboration between the actor and the first author of this paper. The acting performance served as a non-dance control condition and was designed to match the use of the space and bodily actions (clapping, stamping, and hand-fisting) in the two dance pieces. The instrumental ‘one hour in a room’ from ‘Midnight Moth’ (2007) was used as background music. A digital recording of the live performances in the testing space can be seen on http://paco.psy.gla.ac.uk/watchingdance. The performance space spanned 6.8 m in width and 13.5 m in length. In order to capture the whole performance in a straight unedited shoot, we positioned the camera about two meters behind the spectators' viewpoints and used a wide-angle camera lens with a focal length of 5.8 mm to 81.2 mm, which captures the space within a visual angle of 62°. As our participants were exposed to real live performances, their visual angle remained at their maximum potential peripheral vision of 120°. The visual viewing angle when looking at the performer changed during the performance from a maximum of 80.73° to a minimum of 6.04°, dependent on the height of the performers (between 1.62 m and 1.70 m) and their location in space. Importantly, the minimum visual angle was still larger than the human focal vision.

**Figure 1:**
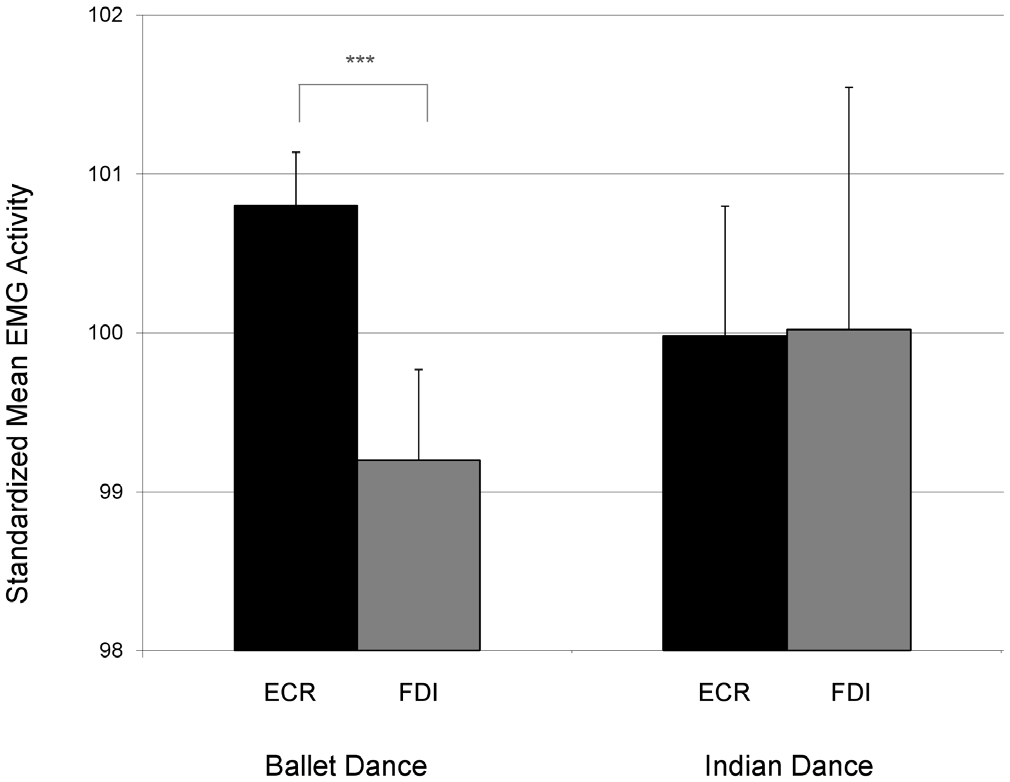
Performers' muscle group activity during the dance pieces. Arm (ECR) and hand (FDI) activity of the ballet and Indian dance performer throughout the whole dance performances. x-axis: ECR (black column) and FDI (grey column) averaged across left and right body side for the ballet performer/ballet performance (left) and Indian dance performer/Indian dance (right). y-axis: standardized mean activities (z-transformed with a mean of 100 and SD of 1 for illustration purposes) compared to overall muscle activity of each performer (See Text S2 for detailed analysis description). *** = significant at *P*<0.001.

The stimuli were evaluated, first by means of a pilot study to test for possible effects of the performers' identity (see Text S1) – which differed between the three conditions – and second by means of comparative analysis of the predicted use of the muscle groups during the different performances (see Figure 1, Text S2 and Text S3 for detailed descriptions and analyses of these evaluations). The pilot study confirmed that the performers' identities *per se* did not have an effect on MEP amplitude, in accordance with brain imaging studies of action observation [e.g. 13]–[14]. Also, the performances in the experiment described here used the muscle groups in the suggested specific manner in our stimuli. At TMS trigger time-points, arm movements were predominant in the ballet performance condition and finger movements were predominant in the Indian dance condition. However, as visible in Figure 1, while arm muscles were used more than finger muscles by the ballet performer in the ballet performance, the Indian dance performer did not activate her finger muscle groups to a greater extent than her arms throughout her performance. In addition, the pilot study showed that MEPs were significantly larger during dance movements than during static dance postures for the more experienced spectators. This result highlighted the importance of measuring cortical excitability during observation of a continuous flow of dance movements.

### Questionnaire

Participants completed an online questionnaire in which they had to rate how visually experienced they had become in watching ballet performances, Indian dance, and theatre plays on 10 point Likert scales (referred to as *visual experience*). The questionnaire further included a standardized empathy questionnaire (Interpersonal Reactivity Index [52]). This test consists of four subscales which assess different aspects of affective or cognitive empathy: (1) *empathic concern* (the tendency to experience feelings of sympathy and compassion for others in need), (2) *personal distress* (the extent to which an individual feels distress as a result of witnessing another's emotional distress), (3) *perspective taking* (the dispositional tendency of an individual to adopt the perspective of another), and (4) *fantasy scale* (the individual's propensity to become imaginatively involved with fictional characters and situations).

### TMS/EMG

Single-TMS monophasic pulses were delivered using a Magstim 200 stimulator (Dyfed, UK) according to standard procedure [53]–[56] through a 90 mm circular coil with anticlockwise current flow, positioned over the vertex which elicited MEPs in both the right extensor carpi radialis (ECR) in the forearm and the right first dorsal interosseous (FDI) in the hand. TMS intensity was set at 120% of the resting motor threshold. The threshold was defined for each participant individually as the lowest TMS intensity that elicited MEPs in the FDI muscle larger than 50 µV in 5 out of 10 stimulations. The mean TMS stimulation threshold did not differ significantly between the three spectator groups (ballet, Indian dance, and novices), 54.00 (8.31 SD), 56.00 (8.07), and 53.11 (6.39), respectively. EMG responses of the ECR and FDI were detected by 8 mm Ag/AgCl sintered flat electrodes in a standard belly-tendon montage. The ground electrode was placed at the elbow joint, over the right lateral epicondyle. EMG signals were recorded in time-windows of between 100 ms before (baseline corrected) and 500 ms after TMS using a customized pre-amplifier (CED 1902) and software (Signal 4.06) with 1000 Voltage gain, 2.5 kHz sampling rate, 20–1000 Hz filter and Notch Filter.

### Procedure

The testing took place in the rehearsal space of the Scottish Ballet company in Glasgow. After the participants had been made familiar with the procedure, the experimenters cleaned their skin at the selected electrode locations with alcohol and applied the electrodes with conductive paste. Participants were asked to wear ear protection. We assured them, however, that they would still be able to hear the music that accompanied the dance performances, even through the ear protection. Participants were invited to simply enjoy the live performances they were going to watch. To minimize the number of runs we tested two participants at a time; they sat next to each other, separated by an occluding panel. The simultaneously tested participants were not always from the same spectator group. The TMS coils were held in place by different experimenters and TMS pulses were triggered simultaneously for both participants using identical equipment. During each performance, 30 single TMS pulses were triggered randomly between 7 and 9 seconds apart. We also measured a series of MEPs when the participants were at rest with their eyes closed, before the live performances (30 trials) and after (15 trials). Immediately after the experiment, individual semi-structured interviews were conducted and participants had to provide the interviewer with the exact number and title/ description of the performances they had seen in the last year. Further, participants were contacted at a later point in time to complete the online custom-designed questionnaire.

### Data Analysis

First, trials with artefact convolution based on technical errors as noted during the testing were excluded (e.g. when participants were moving or when the dancer fell). This affected 3% of all trials, which were equally distributed across participants and conditions. Second, MEP amplitudes (from min peak to max peak within a time window of 10 to 40 ms after the TMS trigger) were measured using a dedicated script written in Matlab (Mathworks, Inc, 2008). Third, we then computed the mean MEP amplitude for each participant for each condition and z-transformed the values in order to achieve a mean of zero and a standard deviation of one across all conditions (i.e. average rest 1 and 2, ballet, Indian dance, and acting control) for each individual participant. Finally, the MEP amplitudes were tested for main and interaction effects using the univariate approach to repeated measures ANOVA in SPSS 18. For these analyses, we subtracted the z-transformed MEP values of the acting control condition from the ballet and the Indian dance MEP values, in order to identify ballet and Indian dance specific responses. Since our groups were unbalanced, we applied the type III sum of squares method in SPSS which is invariant to cell frequencies and equivalent to Yates' weighted-squares-of-means technique. All *t*-test reported were pair-wise and *p*-values were Bonferroni-adjusted for the number of tests conducted. Data values are expressed as mean (M), and standard error (SE) unless stated otherwise.

## Results

### 1) Visual experience

To corroborate group assignment established during pre-screening, the visual experience of participants was assessed after the experiment (in addition to the pre-screening) in two ways: in the form of number of performances seen in the last twelve months and in the form of subjective ratings. The ballet spectators were able to remember on average 13.75 (SD 27.89) performances they had seen in the last year. Indian dance spectators named an average of 4.75 staged live performances (SD 3.24). However, in addition to these formal staged performances, these spectators also reported watching - often more than once a week - other, non-staged semi-professional social performances (e.g. at weddings) and/or performances on screen (e.g. in movies). The number of these social dance events witnessed by Indian dance spectators remains vague. Knowing that reported experiences of dance are always constructed [57], we felt that further inquiring would not enhance the credibility of the responses. Hence, only formally staged performances were included in the analysis of visual experience. Novices did not report watching any named ballet or Indian dance performances. The median number of performances seen each year was the same across the two experienced spectator groups, non-parametric independent-samples median test, df(1), N = 20, Median = 4, asympt. *P* = 0.852.

**Figure 2:**
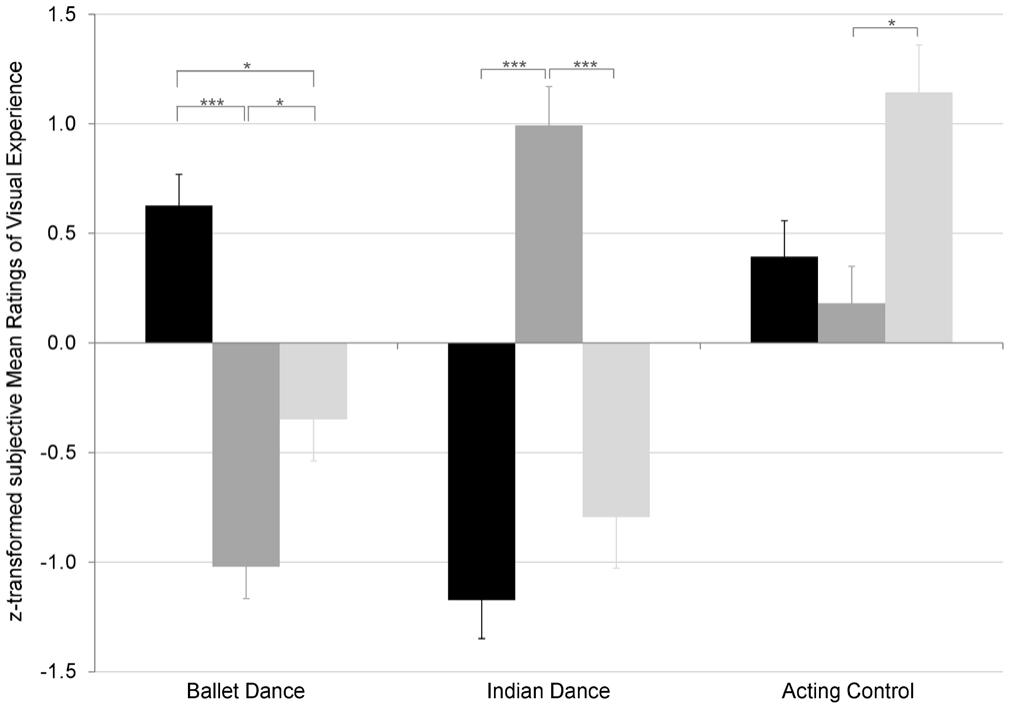
Visual experience for different movement styles. y-axis: z-transformed ratings of visual experience (mean ± SE). x-axis: visual experience of ballet spectators (N = 12) in black columns, Indian dance spectators (N = 9) in grey columns, and novices (N = 8) in light grey columns for different types of performances (ballet, Indian dance, and acting control). The ratings confirmed our experimental groups: the level of acquired visual experience was dependent on the spectator group. *** = significant at *P*<0.001, * = significant at *P*<0.050.

The participants' subjective ratings on how experienced they had become over years in watching ballet, Indian dance, and theatre are displayed in Figure 2 and further confirmed our three spectator groups. A repeated measures univariate analysis of the z-transformed means revealed a significant main effect of the subjective experience ratings for the different performance types, *F* (2, 52) = 11.73, *P*<0.001, a significant main effect for the different groups, *F* (2, 26) = 4.05, *P* = 0.029, and most importantly, a significant interaction between the two factors group and rated experience, *F* (4, 52) = 12.25, *P*<0.001. A total of eleven independent *t*-tests were conducted. These showed that ballet spectators reported significantly higher visual experience in watching ballet than did Indian spectators or novices, *t*(15.27) = 9.64, *P*<0.001 (df corrected for unequal variances) and *t*(19) = 3.95, *P* = 0.011, respectively. Correspondingly, Indian spectators reported having gained significantly higher visual experience in watching Indian dance than did ballet spectators or novices, *t*(18) = 8.17, *P*<0.001 and *t*(15) = 8.23, *P*<0.001, respectively. Novice spectators indicated significantly higher visual experience in watching theatre plays than Indian dance spectators *t*(15) = 3.52, *P* = 0.033, but the subjective reported experience in watching theatre plays between novices and ballet spectators did not significantly differ, *t*(19) = 2.54, *P* = 0.220. Importantly, the reported experience in watching theatre plays did not significantly differ between ballet and Indian dance spectators, *t*(18) = 6.47, *P* = 0.999, ensuring equal (un)familiarity with stage performances besides their preferred dance style. Also, the novice group felt equally inexperienced in watching Indian dance as did the ballet spectators, *t*(19) = 1.01, *P* = 0.999. However, the novice group felt more experienced in watching ballet than did the Indian dance spectators, *t*(11.13) = 4.29, *P* = 0.011 (df corrected for unequal variances). Notably, the reported visual experience of the ballet and Indian dance spectators for their preferred dance form as well as the visual experience for the other dance form did not significantly differ, making them equally experienced in their preferred form and equally inexperienced in the other form, *t*(18) = 1.46, *P* = 0.999 (visual experience in ballet for ballet spectators vs. Indian dance for Indian dance spectators), *t*(18) = 0.71, *P* = 0.999 (no visual experience in Indian dance for ballet spectators vs. in ballet for Indian dance spectators).

### 2) Effects on corticospinal excitability: action observation

**Figure 3:**
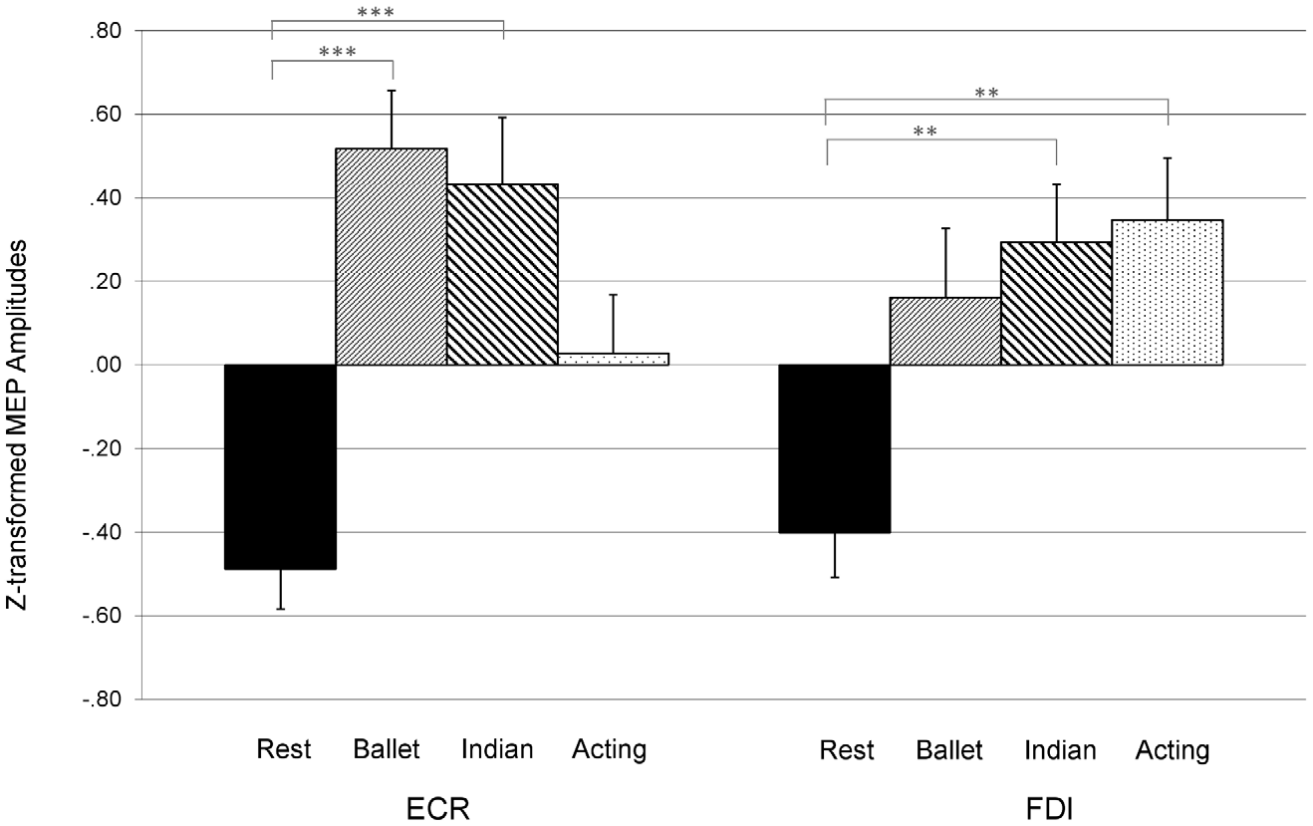
Action observation. y-axis: z-transformed MEP amplitudes (mean ± SE, N = 29). x-axis: rest (eyes closed, black columns), ballet (light stripes), Indian dance (bold stripes), and acting (dots) in ECR (left) and FDI (right). *** = significant at *P*<0.001, ** = significant at *P*<0.010.

When analysing TMS-induced MEPs, we first ensured that no changes had occurred in baseline cortical excitability during the time course of the experiment. The analysis of the within-subjects MEPs of eyes closed before vs. after performances, with the covariate age, showed no significant differences in either location, *F*(1, 25) = 0.41, *P* = 0.841 (FDI), *F*(1, 25) = 0.006, *P* = 0.940 (ECR). For the following analyses, we thus averaged across the two rest conditions. Univariate tests for factor performance (rest, ballet, Indian dance, and acting) and the covariate age showed a significant main effect for performance in the FDI and ECR muscles, *F*(3, 81) = 3.488, *P* = 0.019 (FDI), and *F*(3, 81) = 4.485, *P* = 0.006. The MEP amplitudes showed expected action observation modulation: as visible in Figure 3, z-transformed peak-to-peak amplitude means were lowest in the rest condition. Using a conservative 2-tailed Bonferroni adjusted approach, two out of three contrasts conducted in FDI and ECR reached significance at a 0.05 level. In the ECR, watching dance evoked larger MEPs compared to rest, *t*(28) = 5.48, *P*<0.001 (ballet:rest), and *t*(28) = 4.09, *P* = 0.001 (Indian:rest) while the contrast of acting to rest did not reach significance, *t*(28) = 2.48, *P* = 0.060 (acting:rest). In the FDI, the MEP amplitude difference between watching ballet and rest did not reach significance at a 0.05 level, *t*(28) = 2.42, *P* = 0.067 (ballet:rest), while watching Indian dance or acting led to significantly higher MEPs compared to rest, *t*(28) = 3.38, *P* = 0.007 (Indian:rest), and *t*(28) = 3.34, *P* = 0.007 (acting:rest).

### 3) Effect on corticospinal excitability: visual experience

**Figure 4:**
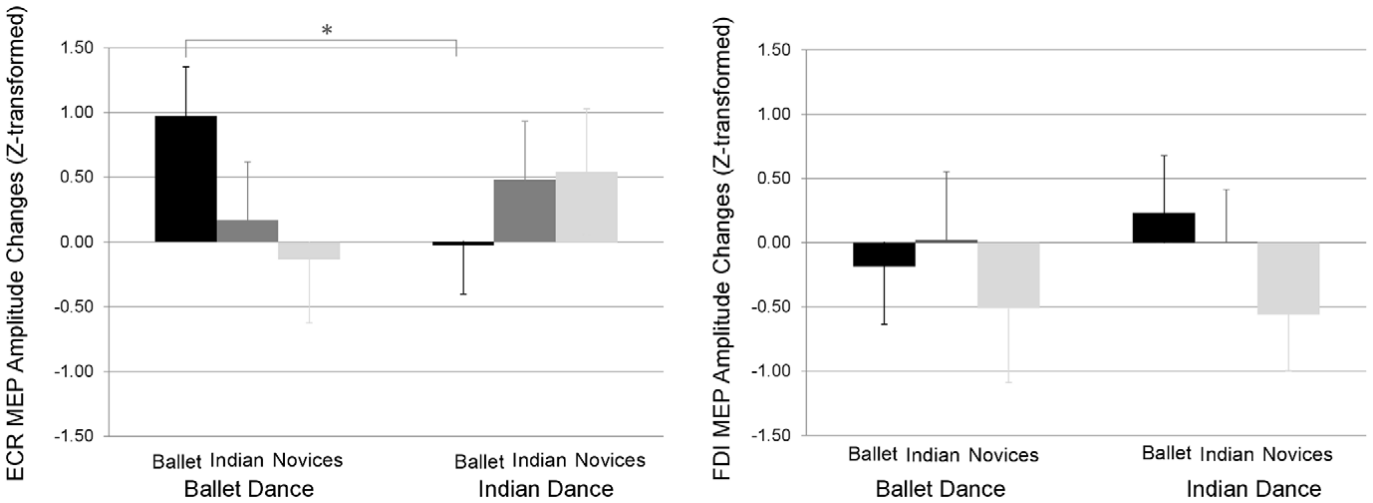
MEP modulation by visual experience. y-axis: MEP amplitudes (z-transformed, mean ± SE, N = 29) of the forearm (ECR, left figure) and hand (FDI, right figure). x-axis: visually experienced ballet spectators (black columns), Indian dance spectators (grey columns), and novices (white columns) during watching ballet specific movements (left) and Indian dance specific movements (right) performances (each as a contrast to non-dance control performance). * = significant at *P*<0.050.

Because we were more interested in motor corticospinal excitability while watching dance-style- specific movements than aspects of general movement observation, we subtracted the mean amplitude of MEPs recorded during the control movement condition (i.e, non-dance control condition) from the mean MEP amplitude recorded during each dance performance, (i.e. ballet or Indian dance) for each participant [see also 11]. We thus analyzed the effect of visual experience (ballet, Indian dance, and novices) on performance specific movements (ballet vs. Indian dance, contrasted with control) on the mean z-transformed MEP amplitudes in ECR and FDI. The univariate within-subjects effects revealed no significant main effect but a significant interaction between performance and visual experience in the ECR, *F*(2, 25) = 5.42, *P* = 0.011. No significant differences were observed in the FDI. We conducted nine *t*-tests to contrast all the differences in the ECR muscle between and within spectator groups when watching ballet or Indian dance specific performances. None of the differences between groups reached significance (e.g. ballet vs. Indian dance spectators when watching the ballet performance). However, the type of dance performance significantly modified ballet spectators' ECR muscle MEPs. As can be seen in Figure 4, experienced ballet spectators showed significantly larger MEPs in the ECR muscle when they watched the ballet performance compared to when they watched Indian dance, paired *t*-test, *t*(11) = 0.47, *P* = 0.018. Novices and Indian dance spectators showed on average smaller MEPs in ECR muscle when they watched ballet compared to when they watched Indian dance, though the differences were not significant, *t*(8) = 0.94, *P* = 0.189 (novices), and *t*(7) = .43, *P* = 0.999 (Indian dance spectators). Thus, the ballet spectators showed a specific response with enhanced corticospinal excitability while watching ballet compared to watching Indian dance.

Because we had participants from different cultural backgrounds and the cultural background has been found to affect cortical excitability during action observation [32]–[33], we conducted the same repeated measures analysis as above but with the between-subjects factor *skin color* in place of visual experience. Using skin color as the between-subjects factor did not reveal any significant main or interaction effects.

### 4) Effects on corticospinal excitability: empathy

The scores for the four subscales of the IRI questionnaire were similar to those originally reported by Davis [52] and were within one standard deviation of the normed mean, namely 21.14 (SD 3.94) for Empathic Concern (EC), 10.90 (5.12) for Personal Distress (PD), 19.17 (4.68) for Perspective Taking (PT), and 16.76 (6.51) for Fantasy Scale (FS). Moreover, none of the subscales differed between the three groups of spectators (ballet, Indian dance, novices), *F*(2, 26) = 1.724, *P* = 0.198 (EC), *F*(2, 26) = .58, *P* = 0.569 (PD), *F*(2, 26) = .84, *P* = 0.445 (PT), *F*(2, 26) = 0.20, *P* = 0.922 (FS). Importantly, when the IRI subscales scores were included as covariates in the repeated measures analysis described above (paragraph 2, Figure 4), the main results of our study remained unaltered. That is, we still observed a significant interaction between the spectators' visual experience and the amplitude of ballet performance specific MEPs in the ECR, *F*(2, 22) = 14.47, *P*<0.001. In addition to this interaction, the repeated measures analysis also revealed a significant effect of the empathy covariate FS on MEPs in the ECR muscle, *F*(1, 22) = 5,61, *P* = 0.027. None of the other factors reached significance at 0.05. Even though the groups did not significantly differ in the average score on any of the four empathy subscales, it is possible that different types of empathy have different effects on motor simulation for different spectator groups when watching dance. To further investigate such group-specific effects of empathy on corticospinal excitability, we calculated Pearson's linear correlation coefficients between each set of scores and MEP amplitudes during watching a dance or acting performance for each group individually. Pearson's correlation showed a significant positive correlation between FS and MEP amplitude in the ECR muscle when Indian dance spectators watched Indian dance, *r*(6) = 0.76, *P* = 0.030 (uncorrected for multiple comparisons). No other correlation reached a significance level of <0.05.

## Discussion

In this paper, we first explored how visual experience in watching certain types of dance movements affects corticospinal excitability while watching live dance performances. Importantly, none of the participants tested reported having had any formal training in any dance style. Thus none of our participants had *motor* experience in the dance movements observed in the study. Yet the participants had specific *visual* experience in either ballet or Indian dance. This was confirmed by their subjective ratings on the level of acquired visual experience in watching ballet or Indian dance. We found that visual experience as well as the Fantasy Scale, a cognitive empathy component of the Interpersonal Reactivity Index, modulated motor resonance in the arm muscles dependent on the performance style. In particular, corticospinal excitability was enhanced in the arm muscles when ballet spectators were watching ballet compared to when they were watching Indian dance and further, corticospinal excitability in the arm muscles was positively correlated with the Fantasy Scale for Indian dance spectators who watched Indian dance. Below we discuss these findings in turn, as well as methodological considerations inherent to using live events as stimuli.

### 1) Effect of visual experience of ballet spectators

The visual experience of ballet spectators was associated with increased MEP amplitudes in the forearm (ECR) but not in the fingers (FDI) while watching the ballet performance. We speculate that ballet spectators were able to selectively simulate those limb movements that are part of the common motor repertoire of the dance style they had experience in watching. In ballet, the fingers are held in a particular shape throughout, whilst the wrist and elbow joints are continuously moved during the dance performance. Clearly, a professional ballet dancer is trained to support their arm movements with their back muscles. However, the arm muscles are still being used more than the finger muscles, as found in our comparative analyses of this specific performance (see Figure 1, Text S2 and Text S3). Therefore, even though visually experienced ballet spectators simulated the movements in a kinematic-compliant manner, we do not claim that their responses fully corresponded to how a professional performer would perform and experience the movements: spectators showed enhanced corticospinal excitability for how “they would perform the movement”, if they had to, and not how an expert would do it. Neither of the other groups of participants, Indian dance spectators or novices, showed any difference in MEP amplitudes between performances.

This discovery extends previous studies which have shown that the brain areas involved in motor planning are more active during action observation when the observer has a motor repertoire for the movements witnessed than when they do not [e.g. 8]–[9], [ 12]–[14]. In our study, we compared different groups of visually experienced spectators who were not trained in executing the movements they were observing; hence, they lacked motor experience. In contrast to Calvo-Merino et al. [9], we were thus able to compare the brain's responses to movements that spectators had acquired visual experience in watching with its response to novel movements, and in contrast to Cross, Kraemer et al. [14], our design prevented an observational confound with practice of a similar task. The finding that experienced ballet spectators showed enhanced corticospinal excitability in their arms (ECR) when watching a live ballet performance compared to a live Indian dance performance could be related to the more frequent occurrence of arm movements in ballet only; but we do not think this is the main cause of our finding. First, if frequency alone modified spectators' responses, then one would expect enhanced corticospinal activity in arm muscle groups in all spectators when watching the ballet performance. Second, repetition of a stimulus during an experiment is more likely to lead to a decrease in activity rather than an increase [58]. Thus it appears that visual experience is responsible for the enhanced direct matching resonance. This finding is in line with observations showing that motor and visual experience can both facilitate motor resonance during action observation [10] as also shown by enhanced corticospinal excitability during action observation [5].

Based on the assumption that enhanced corticospinal excitability during action observation is a marker of resonance in cortical motor circuits, our results suggest that this resonance phenomenon, which possibly relies on mirror-neuron activity, can be established by visual-motor matching. In other words, a ‘personal’ physical knowledge can be acquired indirectly, via visual experience. We do not suppose that the physically inexperienced spectators' sensory experience of the movements fully matched those of the performer. However, our data show that the visually experienced spectators' corticospinal excitability corresponded to the relative muscular activity of the arm and finger of the performer. Hence, visually experienced spectators inherently “mirrored” the observed movements with the proper muscular participation. They ‘understood’ the movements on a neuronal sensorimotor level by having access to the action semantics of those movements for which they had gained visual experience [see 59]. It is possible that the frequent visual exposure shaped the untrained spectators' mirror-neuron system, for example by means of associative learning [see 60] in an indirect way, via their sensory experience of a motor simulation that best approximated the movement observed rather than the execution itself. Indeed, Fecteau et al. [61] found evidence that prolonged visual exposure to an initially neutral stimulus can evoke specific mirror neuron-driving responses in humans. The authors suggested that a direct association between motor practice and perception is not required to develop mirror neuron properties. Other evidence for functional reorganization through internal movement simulation without overt execution has been shown in hemiplegic stroke patients [62]. Further, our data could also be related to results showing that observing hand actions to which a meaning can be attributed enhances left primary motor cortex excitability [63]. In fact, the very essence of dance resides in the choreographed relationship between meaning and movement [41]. In dance, as in any other art form [64], the spectator is cognitively, emotionally, but also aesthetically engaged with the artifact. One can indeed speculate that frequent ballet spectators are more likely to attribute a meaning to the dancers' arm movements than inexperienced spectators. This assumption alone, however, would not explain the muscle specific responses observed here. Altogether, our results suggest that even a ballet audience who lack motor experience in the movements performed on stage show enhanced corticospinal modulation while observing those movements for which they have visual experience. This might be related to their enhanced understanding and enjoyment of watching these movements.

### 2) Effect of empathic abilities

In addition to visual experience, we also found that a specific cognitive empathic ability, assessed by the FS of the IRI [see 52], had a significant effect on MEP modulation. We hypothesized that empathic abilities affect action simulation. This assumption was primarily based on previous studies showing that corticospinal excitability in action observation is modulated by personal dispositions [46]–[47]. These two studies used the Empathy Quotient (EQ) and the Autism Spectrum Quotient (AQ) by Baron-Cohen and co-authors to measure empathy [see 32]. We investigated modulation of corticospinal excitability by means of the IRI in order to further dissociate different components of empathy when watching actions with different levels of relevance for different types of spectators. We found that our effect of FS on MEP modulation is driven by Indian dance spectators; the higher they scored on FS, the larger the MEP amplitudes in the ECR muscle were when watching Indian dance specific movements. The FS characterizes the propensity to identify with fictional characters. At present, the relationship between the activity in the action-observation network and the non-affective cognitive (FS, PT) and emotional (EC, PD) aspects of empathy is not clear. All IRI subscales have so far been found to significantly correlate with some of the brain areas related to the action-observation network and its associated limbic structures [e.g. 65]–[68]. During the observation of penalty shoots, for instance, emotional empathy in the form of PD has been shown to correlate positively with anterior cingulate cortex (ACC) activity while EC was found to correlate negatively with the supplementary motor area and ACC activity [68]. Cognitive empathy (FS, PT) was found to be correlated with brain areas activated in observing painful stimuli [65]. Further, emotional (EC, PD) and cognitive subscales (FS) correlated significantly with activity in the inferior frontal mirror neuron area during observation of precision grips [67]. Based on these previous findings, we had no specific prediction as to which empathy subscale would show a significant modulation of corticospinal activity in our study. Our finding showed that corticospinal excitability was enhanced for those Indian dance spectators who scored higher in FS – and may have been more likely to be engaged in the narrative of the performance.

Notably, we did not find an effect of cognitive empathy on corticospinal activity in the finger muscles. The use of these muscles distinguishes the Indian dance performance from the ballet performance, namely that the fingers are articulately miming different everyday actions, such as eating or playing the flute in the Indian dance, whereas in the ballet performance, it is the arms that are bending or stretching to serve the goal of the action. If cognitive empathic abilities could compensate for the lack of motor experience and thereby modulate spectators' mirror-neuron system on a neuronal sensorimotor level, one would also expect the FDI to be significantly enhanced with increased FS when watching Indian dance. The correlation between FS and MEP amplitude was significant when watching the Indian dance but in the ECR muscles only, suggesting that movements were not mirrored in a dance style specific manner. This finding extends previous studies, in that individuals with high cognitive empathy may automatically engage with the emotional states of others [e.g. 64] but not in a muscle specific manner. It is thus reasonable to conclude that not only do different aspects of empathy depend on different neural substrates, as suggested by Gazzola, Aziz-Zadeh, and Keysers [66], but that measures from different empathic subscales relate differently to the modulation of motor resonance depending on the stimuli and their context [e.g. 67].

Notably, the recognition of most Hindu specific emotional expressions has been shown to be universal [69], in contrast to ballet which is more formal. We suggest that visual experience can potentially modify corticospinal excitability during observation of formal movements such as those in ballet, while interpersonal skills can eventually modify corticospinal excitability during observation of gestural actions such as those in Indian dance. Interestingly, this was only significant for Indian dance spectators, who are more familiar with Hindu expressions than novices or ballet spectators. In other words, different types of motor simulation may exist, dependent on whether simulation is based on kinesthetic resonance formed through visual (or motor) experience or whether simulation is based on specific factors of empathy. Here, the cognitive empathy factor FS did only play a significant role as a modifier of corticospinal excitability when watching a particular dance form (i.e. Indian dance in our study) which the spectators were visually familiar with. Motor simulation may indeed be evoked via different routes, with or without mirror neuron reliance. Buccino et al. [70], for instance, suggested that actions can be recognized by motor properties via a spectator's motor repertoire or in non-motor terms by visual properties. In addition, Van Overwalle and Baetens [71] suggested that the mirror-neuron system could be linked with the mentalizing system, which is thought to be activated when actions are presented in abstract terms. The authors suggested that the two systems have never been found to be concurrently active because most experiments have been conducted under strictly controlled laboratory settings. Further studies that allow multilayered processes in action observation are thus needed to validate whether these two means of motor simulation with or without mirror-neuron reliance are independent from each other, and how they relate to the different means of action understanding.

### 3) On using real-life events as stimuli

Importantly, this study showed that it is possible to collect valid data using a *real life event* with high ecological validity. Although reductionist approaches have much to offer [72], the appreciation of art is notably influenced by context [73]. One such important contextual aspect of our study was the use of continuous flow of movement. While dynamic information can be extracted from static images [74]–[75], our pilot study (see Text S1) showed that effects of expertise are most likely to be responsive to continuous moving bodies that match the spectators' actual visual experience; in our case, a live dance performance. Nevertheless, the high ecological validity of our experiment led to a number of restrictions and implications, as discussed below.

In particular, we expected that the visually experienced Indian dance spectators would show enhanced corticospinal excitability in the FDI when watching Indian dance compared with the other groups. We believe that the absence of an effect here is due to the balancing act of designing a scientific experiment and the occurrence of the factors in real life. First, the performers did not employ the FDI and ECR in their dance as we had predicted; the ECR was significantly more highly activated by the ballet dancer throughout the ballet performance than by the Indian dancer in the Indian performance, as we expected. However, the reverse was not the case; namely, the FDI was not significantly more activated by the Indian dancer throughout the Indian dance performance than by the ballet dancer throughout the ballet performance, despite the importance of finger signs in the Indian dance.

Further, the participants' visual experience has been modified by the cultural background in which they live. For instance, visually experienced ballet spectators who are not trained in ballet are generally above the average age of participants usually included in Psychology experiments. In addition to this, we found that novices were more likely to report being visually experienced in watching ballet performances than Indian dance spectators, and similarly, both ballet spectators and novices were more likely to report being visually experienced in watching theatre plays than Indian spectators. It may be the case that the novices and ballet spectators were more integrated into the Western lifestyle than the Indian dance spectators, and thus were more often exposed to ballet and theatre performances. However, experienced Indian dance spectators reported that watching Indian dance is integrated in their everyday life (primarily on TV, but also informal, social experiences of Indian dance) - though this is a broad category of Indian dance, rather than the Bharatanatyam used in our study. In contrast, ballet spectators specifically seek out live ballet performances, ballet performances on film, and on television. Importantly, to Indian dance experts the screen experience was not seen as inferior to the live experience in the same manner in which it was for ballet watchers. Hence, one key factor remained consistent; the ballet spectators did not report watching ballet significantly more often than the Indian dance spectators reported watching Indian dance. Nevertheless the type of performance may have differed; Indian dance spectators reported attending a smaller number of staged performances. This is purely due to cultural differences (ballet is not normally performed at parties, as the Indian dance often is) but the visual experience may thus be less specific for Indian than ballet spectators and potentially be better described as visual familiarity. The slightly reduced level of expertise of the Indian dance spectators in Indian dance compared with the ballet spectators' experience in watching ballet was a recurrent theme in the qualitative audience research [57], which was conducted alongside this quantitative experimental study [see also 76]. The effect of the spectators' cultural background has also recently been discussed in other TMS studies on action observation. Désy and Théoret [32] found enhanced MEPs when females observed hand actions performed by actors of different skin color or gender, whereas Molnar-Szakacs et al. [33] found enhanced MEPs when Euro-American spectators watched gestures performed by a Euro-American actor. Because the pleasantness of visual images has also been shown to alter corticospinal excitability [77]–[79], we were specifically interested in measuring the effect of visual experience on corticospinal excitability when watching dance in its typical culturally embedded occurrence. Our ballet and acting performers were thus white European while our Indian performer was a non-white UK resident with an Indian-Tamil background. Despite the difference in skin color congruence between our spectators and performers, we did not find any significant effect of skin color on corticospinal excitability. Based on our findings, we conclude that visual experience in its situated cultural form enhances fine-tuned motor simulation.

### 4) Effect of music

We purposely chose to accompany each performance with the commonly associated music in order to enhance ecological validity by creating a performance that matched the experience of most dance performances. In dance, kinesthetic aspects, movement expression and the meaning of actions are intertwined with the music, costumes, lights, and stage settings. The response to watching dance, then, is evoked by the whole range of visual-auditory stimulation and is thus not a response to movement only. Theoretically, one could argue that corticospinal excitability could have been modified by the music rather than the movements. Alaerts, Swinnen, and Wenderoth [80] observed convergence of the sound and sight of hand actions as a generic feature of the mirror-neuron system recruitment. Here, we were interested in the effect of visual experience of watching dance, which includes enjoyment, conveyance of meaning, and emotion. This was measured by the effect of live dance performances on corticospinal excitability. McNamara et al. [81] demonstrated that the neuronal circuits of the motor system are involved in learning novel sound-action associations. Hence, for the experienced dance spectators, the response to watching dance is supposedly associated with the whole multisensory experience, in which all the strands of dance, in particular movement, sound and music, play a part. Experienced dance spectators go and watch dance – otherwise they would, for example, only be experienced music spectators. This is why we refer to the spectators' responses to dance rather than to movement. Thus, we say that dance in its best form allows the spectator to engage with the dancer, and induces ‘specific motor simulation’. The extent to which the presence or absence of music affects the spectators' responses will be the subject of one of our future investigations.

### 5) Conclusion

To conclude, we have provided evidence that both visual experience and empathic abilities can increase motor resonance with the observed movements. Thus, the motor repertoire of the spectators is not the sole factor that modulates the neurophysiological response to watching dance. We propose that motor simulation in action observation is possible via two pathways. As suggested previously, motor simulation can be driven by a direct motor resonance, resulting in kinematic congruency of observed and simulated movements. Further, motor simulation may occur by indirect action generation as a result of cognitive empathic abilities. In the latter case, a kinematic match between observed and simulated action is not necessary. Future studies are required to investigate the level of visual experience needed for direct motor matching responses, and how stable they are over time.

## Supporting Information

Text S1 **Supplementary materials.**
(DOC)

Text S2 **Supplementary materials.**
(DOCX)

Text S3 **Supplementary materials.**
(DOC)
