# Supplementary material for: Motor Simulation without Motor Expertise: Enhanced Corticospinal Excitability in Visually Experienced Dance Spectators
Source: PLoS One. 2012 Mar 21;7(3):e33343. doi: 10.1371/journal.pone.0033343 (PMC3310063; doi:10.1371/journal.pone.0033343)
Supplement: Text S1 — Supplementary materials. (DOC) [file pone.0033343.s001.doc]

**S1. Pilot study: Effects of performer identity.** Since the performer’s body and movement style in dance are always intrinsically intertwined, we conducted a pilot study to test for effects of the performer. We tested corticospinal excitability of the FDI and ECR muscle groups of six subjects who had different levels of experience in watching western dance style movements. Subjects watched two performers each staging a dance performance, as well as two sets of 24 still postures extracted from the dance performances. This resulted in two conditions: dance performer (Indian dancer vs. ballet dancer) and movement (dance postures vs. dance movements). Both the dance movements and the postures were performed live. The performers held each of 24 static frame postures, which were selected from the dance movements, for at least 4 seconds. Data processing and analysis were carried out as described in the main article. Univariate analysis with spectators’ visual experience in watching dance as an additional covariate variable showed that z-transformed MEP amplitudes did not show a significant main effect of dance performer on MEP amplitudes, *F*(1, 4) = 2.06, *P* = 0.225 (FDI), *F*(1, 4) = 0.13, *P* = 0.730 (ECR). With no main effect of dance performer, we could rule out the assumption that the mere presence of a particular dancer had an effect on corticospinal excitability in FDI or ECR muscle groups. However, we found a main effect of movement in FDI but not in ECR MEP amplitudes, *F*(1, 4) = 25.24, *P* = 0.007 (FDI), *F*(1, 4) = 0.31, *P* = 0.605 (ECR). Based on the significant interaction expertise and movement in FDI, *F*(1, 4) = 24.89, *P* = 0.008, we conducted a correlation analysis. The more experienced the spectator, the larger the MEP amplitudes in response to watching movements, pearsons’ *r*(4) = 0.928, *P* = 0.008. This pilot study demonstrated two important points: First, FDI MEP amplitudes were significantly higher while observing dance movements compared to stills; the more visually experienced spectators showed that it is relevant to measure the response to movements rather than postures, especially when testing visual expertise. Second, the ECR MEP amplitude was not significantly higher for movement than for stills, which we assigned to the low level of the performance of the ballet dancer. We thus engaged a different, more professional ballet dancer in the main study.
