# Supplementary material for: Motor Simulation without Motor Expertise: Enhanced Corticospinal Excitability in Visually Experienced Dance Spectators
Source: PLoS One. 2012 Mar 21;7(3):e33343. doi: 10.1371/journal.pone.0033343 (PMC3310063; doi:10.1371/journal.pone.0033343)
Supplement: Text S2 — Supplementary materials. (DOCX) [file pone.0033343.s002.docx]

**S2. Specific use of muscle groups in ballet and Indian dance.** We measured the EMG activity of the performers’ arm and hand muscles during the whole performance in a separate session. This measure allows a quantitative comparison between the use of arm and hand muscles for both dance styles.

The mean rectified ECR and FDI muscle activity of the two performers showed that the mean ECR activity was higher in ballet than in the Indian dance, and the FDI was higher in the Indian dance than in ballet, as can be seen in Figure 1 in the main document. To test for significant differences, EMG was rectified and averaged across time windows of 500 ms and a set of 30 exemplars were selected randomly for each arm and each performer. Each performer’s values were standardized across their four measures, FDI left, FDI right, ECR left, and ECR right, in order to allow a comparison between present muscle activities and dance specific overall activity. Paired *t*-tests with Bonferroni adjusted *p*-values showed that muscle activity in mV was significantly higher in ECR than FDI in the ballet performer, 0.048 (SD 0.026) vs. 0.019 (0.008), *t*(29) = 7.47, *P* < 0.001 (left side); 0.047 (0.017) vs. 0.025 (0.013), *t*(29) = 7.72, *P* < 0.001 (right side). Unexpectedly, however, the difference between FDI and ECR was not significant in the Indian performer, 0.045 (0.023) vs. 0.052 (0.023), *t*(29) = 1.20, *P* = 0.477 (left side); 0.058 (0.031) vs. 0.063 (0.021), *t*(29) = .65, *P* = 0.999 (right side).
