# Supplementary material for: Motor Simulation without Motor Expertise: Enhanced Corticospinal Excitability in Visually Experienced Dance Spectators
Source: PLoS One. 2012 Mar 21;7(3):e33343. doi: 10.1371/journal.pone.0033343 (PMC3310063; doi:10.1371/journal.pone.0033343)
Supplement: Text S3 — Supplementary materials. (DOC) [file pone.0033343.s003.doc]

**S3. Muscle group involvement at TMS trigger time-points.** TMS triggers were applied with a random jitter during the performance. We measured the frequency of ECR and FDI muscle group involvement in the movements performed at the time-point when the TMS was applied, in order to identify the actual involvement of these muscle groups in the trials. This was possible as the experimental sessions were digitally recorded on video for all but one subject (Indian spectator). Muscle groups were defined as active when the angle of the hand to the arm (ECR) or the thumb to the hand (FDI) of the performer deviated in either direction from the neutral position. By using this post-hoc qualitative description of the movements performed by the dancer at the moment of the TMS pulses, relative contributions of the muscle groups could be compared.

We conducted paired *t*-tests with Bonferroni adjusted *p*-values of the relative ECR and FDI involvement in percentages (trials with muscle group activity out of total trials per condition) at each TMS trigger time point during the performances. They showed that the ECR was activated significantly more frequently than the FDI during the ballet performances for all groups, 88.98 (*SD* 8.11), 47.75 (16.96), *t*(27) = 15.51, *P* < 0.001, and the ECR was activated significantly less frequently than the FDI during the Indian dance performances for all groups, 70.32 (12.02), 91.83 (7.14), *t*(29) = 10.32, *P* < 0.001. This was also consistent within groups: we observed consistently higher occurrences of ECR than FDI (in percentages) when watching ballet; 91.03 (7.92) vs. 46.26 (14.39), *t*(11) = 15.00, *P* < 0.001 (ballet spectators); 92.50 (7.07) vs. 62.91 (7.45), *t*(7) = 10.39, *P* < 0.001 (Indian spectators); 82.38 (5.84) vs. 34.81 (16.46), *t*(7) = 7.67, *P* < 0.001 (novices); and lower ECR than FDI when watching Indian dance; 72.50 (9.96) vs. 91.67 (7.45), *t*(11) = 6.80, *P* < 0.001 (ballet spectators); 75.00 (5.35) vs. 96.25 (5.18), *t*(7) = 10.70, *P* < 0.001 (Indian spectators); 62.36 (5.82) vs. 87.64 (6.33), *t*(7) = 4.41, *P* = 0.024 (novices).
